# Revisiting hypocholesterolemia during prolonged sepsis: From targeted cholesterol repletion to unresolved adrenal and muscle dysfunction

**DOI:** 10.1016/j.mce.2026.112848

**Published:** 2026-10

**Authors:** Lauren De Bruyn, Fien Van Beek, Sarah Vander Perre, Sarah Derde, Inge Derese, Lies Pauwels, Greet Van den Berghe, Lies Langouche

**Affiliations:** Clinical Division and Laboratory of Intensive Care Medicine, Department of Cellular and Molecular Medicine, KU Leuven, Leuven, 3000, Belgium

**Keywords:** Sepsis, Hypocholesterolemia, Adrenal insufficiency, Muscle weakness

## Abstract

**Background:**

Sepsis HDL- and LDL-hypocholesterolemia associate with adverse outcomes, but whether this necessitates supplementation or merely reflects disease severity remains unclear. We hypothesize that sustained hypocholesterolemia can contribute to ICU-acquired weakness and adrenal dysfunction, and that cholesterol supplementation can improve tissue cholesterol availability, muscle and adrenal integrity.

**Methods:**

In a catheterized mouse model of cecal-ligation-and-puncture-induced sepsis (5-days), septic mice received continuous infusion with an LDL- (mouse study 1; n = 51) (3.5 mg/d) or HDL-cholesterol (mouse study 2; n = 47) (5 mg/d) enriched cholesterol mixture, compared to placebo, and healthy reference mice. Plasma HDL-, LDL-cholesterol, CORT, TNF-α and total bile acids were measured, in addition to muscle force, myofiber cholesterol, *ex-vivo* adrenal ACTH response, adrenal cholesterol and structure. Gene expression markers of cholesterol synthesis were measured in liver, adrenal and muscle tissue.

**Results:**

LDL-cholesterol infusion in septic mice increased plasma LDL-, but not HDL-cholesterol, whereas HDL-cholesterol infusion increased plasma HDL- and LDL-cholesterol (P < 0.0001 versus placebo). Cholesterol supplementation attenuated sepsis-induced adrenal cholesterol depletion (P < 0.05), without improving adrenocortical structure or the adrenal ACTH response. Cholesterol supplementation did not affect muscle mass loss, force or myofiber cholesterol, but increased plasma bile acids and reduced markers of cholesterol synthesis in liver, adrenal and muscle versus placebo (P ≤ 0.05). No additional effect on elevated plasma CORT and TNF-α was observed.

**Conclusion:**

Cholesterol supplementation reversed sepsis-induced hypocholesterolemia, without reversing the sepsis-induced adrenal or muscle phenotype. Together with suppressed markers of cholesterol uptake, synthesis and increased bile acid formation, these findings argue against the need to treat hypocholesterolemia during prolonged sepsis.

## Highlights

• Cholesterol infusion reversed sepsis hypocholesterolemia in prolonged septic mice.
• Cholesterol infusion did not affect the sepsis-induced adrenal and muscle phenotype.
• Cholesterol infusion suppressed cholesterol synthesis in liver, adrenal and muscle.
• These findings argue against treating hypocholesterolemia in prolonged sepsis.

## Graphical abstract

**Figure:**
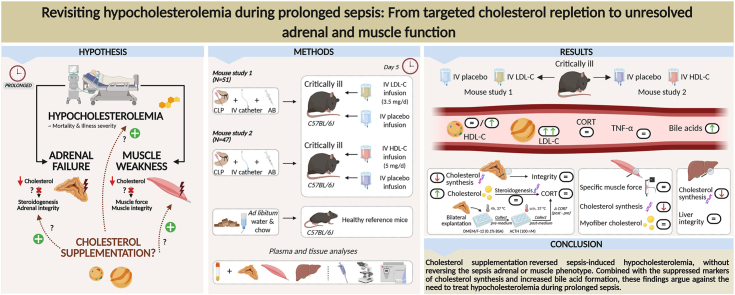

## 1 Background

Cholesterol is vital for key physiological processes including membrane integrity, signaling and immune function, and serves as the precursor for steroid hormones, vitamin D and bile acids. Alterations in the cholesterol availability have been observed in severe diseases and are associated with adverse outcomes (Hofmaenner et al., 2023; Tanaka et al., 2017; Lauwers et al., 2023). In sepsis and other critically ill conditions, plasma concentrations of total, low-density lipoprotein (LDL-) and high-density lipoprotein (HDL-) cholesterol acutely decrease and remain low during disease progression (Hofmaenner et al., 2023; Tanaka et al., 2017; Mesotten et al., 2004; Chiarla et al., 2010; Bakalar et al., 2003; Giovannini et al., 1999; Coombes et al., 1980). This robust decrease in circulating cholesterol is proportionate to the severity of inflammation and illness and is associated with risk of death (Hofmaenner et al., 2023; Dunham et al., 2003). Although the concept of hypocholesterolemia of critical illness is well-described, its underlying pathophysiology and clinical implications are largely unknown. Whether hypocholesterolemia should be interpreted as merely a clinical marker of illness severity or warrants supplementation requires further investigation.

Cholesterol or lipoprotein supplementation in sepsis are interventions that have gained increasing interest, although translation to the clinic has been challenging, likely due to variability in purity of the supplements and in degree of endotoxin contamination, as well as to species-specific differences in cholesterol metabolism (Yin et al., 2012). In preclinical studies, infusion of (reconstituted) HDL-cholesterol or apolipoprotein A1 (ApoA1) has shown to improve organ function and/or survival in acute sepsis and endotoxemia models with limited ICU-supported care (Moreira et al., 2014; McDonald et al., 2003; Tanaka et al., 2020; Kleyman et al., 2025). However, therapies mimicking the endotoxin scavenging aspects of cholesterol, such as treatment with a phospholipid emulsion or with polymyxin B hemoperfusion did not improve outcome (Dellinger et al., 2018), and intravenous administration of a lipid emulsion containing soybean oil, medium-chain triglycerides, and olive and fish oil failed to stabilize cholesterol levels in septic patients (Guirgis et al., 2019; Walley, 2024).

We postulate that sustained low systemic cholesterol availability could become disadvantageous especially in the prolonged phase of critical illness, as it could result in impaired cholesterol delivery to peripheral tissues. The response to stress of the adrenal cortex in particular could become impaired. Indeed, the cortisol response to injection of adrenocorticotropic hormone (ACTH) has shown to correlate positively with plasma cholesterol concentrations in critically ill patients (van der Voort et al., 2003). In addition, a structural impairment and esterified cholesterol depletion have been observed in adrenal glands from long-stay ICU patients, together with reduced expression of key genes driving steroidogenesis (Boonen et al., 2014). These observations suggest that hypocholesterolemia could play a role in the development of intensive care unit-acquired adrenal insufficiency (ICU-AI), a hypothesis that has not yet been tested. Prolonged critically ill patients are also at risk of developing ICU-acquired weakness (ICUAW), a debilitating complication that could be related to a shortage of cholesterol. Serum cholesterol concentrations have been shown to correlate inversely with the severity of muscle weakness in patients with ICUAW and in our mouse model of prolonged sepsis-induced critical illness (Goossens et al., 2021). Remarkably, in sepsis-induced critically ill mice, supplementation with the ketone body, 3-hydroxybutyrate (3HB), which can serve as a substrate for cholesterol synthesis (Hasegawa et al., 2012; Geelen et al., 1983; Lopes-Cardozo and Klein, 1985; Robinson and Williamson, 1980), was found to increase myofiber cholesterol content and to attenuate muscle weakness (Goossens et al., 2019, 2021).

In this study, we investigated whether supplementation with a cholesterol mixture containing predominantly either LDL- or HDL-cholesterol has the ability to improve critical illness-related hypocholesterolemia, thereby potentially affecting the sepsis-induced adrenal and muscle phenotype. Our hypothesis was tested in two consecutive animal studies using a clinically validated mouse model of sepsis-induced critical illness that mimics the human phenotype of the sustained decrease in plasma cholesterol (Goossens et al., 2019), as well as ICU-AI (Teblick et al., 2021, 2022) and ICUAW (Goossens et al., 2017, 2019, 2021).

## 2 Methods

### 2.1 Mouse studies

Male 24-week-old C57BL/6J mice were randomized to two interventional sepsis groups or a healthy control group. Mice in the sepsis groups were anaesthetized with a xylazine/ketamine mixture and received a central catheter in the jugular vein, followed by cecal ligation and puncture to induce sepsis (Derde et al., 2017). In a first study (mouse study 1), septic mice (Charles River Laboratories, Saint-Germain-Nuelles, France) received continuous intravenous infusion with either placebo (NaCl) or with a cholesterol mixture containing predominantly LDL-cholesterol (3.5 mg/d) (bovine source, 185-10, Lee Biosolutions). In a second consecutive mouse study (mouse study 2), with a same experimental setup, septic mice (Janvier Labs, Le Genest-Saint-Isle, France) received continuous intravenous infusion with either placebo (NaCl) or with a cholesterol mixture containing predominantly HDL-cholesterol (5 mg/d) (human source, 0.2 μm filtered (361-10, Lee Biosolutions)). After surgery, septic mice received 24-h intravenous fluid resuscitation (Plasmalyte, Baxter®, containing 4.2% glucose) (0.3 mL/h), whereafter continuous intravenous parenteral nutrition (Olimel N7E, Baxter, Lessines, Belgium) (0.2 mL/h) and cholesterol infusion (0.033 mL/h) were given. In addition, septic mice received twice daily subcutaneous injections with broad-spectrum antibiotics (Imipenem/Cilastin, Fresenius Kabi) and analgesics (Buprenorphine (Vetergesic), Ecuphar, Oostkamp, Belgium). Control mice that did not undergo any procedure and that received *ad libitum* water and standard chow served as a healthy reference group. All animals were euthanized after a five-day study period by cardiac puncture, whereafter blood and tissue samples were collected. A total number of 15 surviving animals per group was required to be able to detect a 20% increase in specific maximal muscle force and a 20% increase in adrenal cholesterol content between placebo and cholesterol supplemented mice, calculated with an α-error <0.05 and >80% power. For mouse study 1, 51 animals survived until the end of the study period: healthy control group, n = 14/14; placebo-infused sepsis, n = 18/22; LDL-infused sepsis, n = 19/22). For mouse study 2, 47 animals survived until the end of the study period: healthy control group, n = 16/16; placebo-infused sepsis, n = 16/19; HDL-infused sepsis, n = 15/24). An additional mouse study (mouse study 3) was performed with the same experimental setup as mouse study 1 to assess the adrenal corticosterone (CORT) response after ACTH stimulation. Calculated with an α-error <0.05 and 80% power, and based on previous data from our lab, 7 animals per group would suffice to detect a normal increase in CORT after ACTH stimulation (De Bruyn et al., 2024). For mouse study 3, 23 animals survived until the end of the study period: healthy control group, n = 6/6; placebo-infused sepsis, n = 9/12; LDL-infused sepsis, n = 8/8). Any dropout during the experiment was attributed to perioperative death, technical failure or functional catheter loss.

All animals were kept in a temperature-controlled (27°C) animal cabinet with 12 h light and dark cycles. Pain/discomfort was assessed twice daily based on the Mouse Grimace Score (Derde et al., 2017; Langford et al., 2010) and cumulative pain scores were calculated to assess illness severity. All animals were treated according to the Principles of Laboratory Animal Care (US National Society of Medical Research) and to the European Union Directive 2010/63/EU concerning the welfare of laboratory animals. All animal procedures were approved by the Institutional Ethical Committee for Animal Experimentation (P098-2021) and complied with the essential 10 ARRIVE guidelines (Percie du et al., 2020).

#### 2.1.1 Plasma and tissue analyses – mouse study 1 and mouse study 2

##### 2.1.1.1 Ex vivo muscle force measurements

Immediately after euthanasia, the hindlimb extensor digitorum longus (EDL) muscle was isolated and placed in a temperature-controlled (30°C), continuously perfused organ bath (95% O_2_, 5% CO_2_) filled with HEPES-buffered Krebs-Ringer solution (0.57 mM MgSO_4_, 10 mM glucose, 4.5 mM KCl, 120 mM NaCl, 0.7 mM Na_2_HPO_4_ dibasic, 0.9 mM Na_2_H_2_PO_4_ monobasic, 5 mM MgCl_2_, 1.2 mM KH_2_PO_4_, 2 mM CaCl_2_, 10 mM HEPES, pH = 7.3) to measure absolute and specific muscle force *ex vivo* (300C-LR Dual-Mode muscle lever, Aurora Scientific), as previously described (Weckx et al., 2021). Absolute tetanic muscle force was calculated as the average of three consecutive tetanic stimuli (180 Hz stimulation frequency). Specific muscle force was calculated by dividing the absolute tetanic muscle force by the calculated muscle cross-sectional area.

##### 2.1.1.2 Plasma analyses

After euthanasia, plasma HDL- (DZ129A-K, Diazyme Laboratories) and LDL-cholesterol (DZ128A-K, Diazyme Laboratories), TNF-α (MHSTA50, R&D Systems, Minneapolis, MN, USA), CORT (EIA5186, DRG International Inc., Springfield, NJ, USA) and total bile acid (DZ042A-KY1, Diazyme Laboratories) concentrations were measured with commercially available assays, as per manufacturer's instructions.

##### 2.1.1.3 Quantification of mevalonate pathway metabolites

In the gastrocnemius muscle, metabolites of the mevalonate pathway (mevalonic acid, isoprenoids, dolichols, ubiquinone, sterols and cholesterol) were quantified with targeted liquid-chromatography mass spectrometry (LC-MS) (TMIC, Edmonton, Canada). Concentrations of the compounds detected in the samples were calculated by interpolating the constructed linear-regression calibration curves using internal or external standard calibration. MRM/MS did not separate isopentenyl pyrophosphate (IPPP) and dimethylallyl pyrophosphate (DMAPP); their total amount in each sample was reported. Concentrations of polyprenols and dolichols were measured as the total weight of all the detected compounds (μg/g tissue).

##### 2.1.1.4 Gene and protein tissue expression

Total RNA was isolated from adrenal gland with the Macherey-Nagel Nucleospin RNA kit (Macherey-Nagel, Düren, Germany) and with the RNeasy mini RNA kit (Qiagen, Hilden, Germany) in gastrocnemius and liver tissue using according to the manufacturer's instructions. Subsequently, RNA was reverse-transcribed into complementary DNA using random hexamers (Invitrogen, Waltham, MA, USA). Commercial TaqMan® assays (Applied Biosystems, Carlsbad, CA, USA) were used for all gene expression analyses. Relative gene expression was determined using the 2-ΔΔCt method, with *Sdha*, *Gapdh* or *Rn18S* as housekeeping gene, and expressed as median log2 fold changes relative to the healthy control group. Gene expression data are presented as heatmaps, created with R statistical software (version 4.4.3, The R Project for Statistical Computing, Vienna, Austria) and the RStudio interface (version 2025.05.0), using the Pheatmap package.

For plasma protein expression of ApoA1 and ApoB100, Western blot analyses were performed. Plasma samples were diluted 1:250 in sample buffer and following homogenization buffer: 1% Nonidet P-40; 10% glycerol; 20 mM Tris-HCl (pH = 7.6); 10 mM EDTA; 1x Complete Mini, and 10 μl was loaded on a 4-20% tris-glycine polyacrylamide gel. Proteins were subsequently electroblotted on a polyvinylidene fluoride (PVDF) membrane, blocked in 5% bovine serum albumin (BSA) and overnight incubated at 4°C with primary antibodies against anti-mouse ApoA1 (ab300085, Abcam; RRID: AB_3720350) (1:250) and anti-mouse ApoB (ab312318, Abcam; RRID: AB_3720351) (1:250). Afterwards, immunoblots were incubated with a horseradish peroxidase- (HRP) linked secondary antibody (Dako, Glostrup, Denmark) at room temperature for 1 h. Immunoblot images were auto-exposed and visualized using chemiluminescence plus technology (PerkinElmer, Zaventem, Belgium) with the G:BOX Chemi XRQ (SynGene, Cambridge, United Kingdom) and images were analyzed with SynGene software (RRID:SCR_022505). Data were corrected for equal loading between gels and presented as fold change of the median of the healthy controls.

##### 2.1.1.5 Adrenal cholesterol content

Whole adrenal glands were frozen in Tissue-Tek (Sakura Finetek) and cut at 8 μm thickness with a microtome-cryostat. Adrenal sections were stained overnight in 0.5% Oil-Red-O (ORO) (Sigma-Aldrich) in propylene glycol and counterstained with hematoxylin. Adrenocortical cholesterol content was estimated as the relative amount of redness in the adrenal cortex with ImageJ 53t (RRID:SCR_003070). Images were taken with a TissueFAXS i PLUS microscope (TissueGnostics, Vienna, Austria).

##### 2.1.1.6 Histology and immunostaining

Cryo-embedded adrenal gland tissue sections were cut at 8 μm thickness and stained with hematoxylin and eosin to investigate structural integrity. To evaluate structural integrity, a ‘0’ score was given when the three adrenocortical zones were clearly distinguishable and displayed a normal, radial, cord-like architecture. A ‘1’ score was given when the adrenocortical zones were moderately distorted and a ‘2’ score was given when the three adrenocortical zones were severely distorted in the presence of extensive tissue damage.

To assess liver integrity histologically, hematoxylin and eosin stained formalin fixed 5-μm paraffin liver sections were semi-quantitatively scored for loss of structure (Jenniskens et al., 2018). A score of ‘0’ (normal structure), ‘1’ (moderately distorted) or ‘2’ (severely distorted) was given to assess liver structure. Tissue-sections were semi-quantitatively scored, blinded for randomization. All images were taken with a TissueFAXS i PLUS microscope (TissueGnostics, Vienna, Austria).

#### 2.1.2 Tissue analyses – mouse study 3

##### 2.1.2.1 Ex vivo adrenal CORT response to ACTH

An additional mouse study (n = 23) was performed with the same experimental setup as mouse study 1 to assess the adrenal CORT response after ACTH stimulation using an *in vitro* adrenal explantation assay, as previously described (De Bruyn et al., 2024). In brief, both adrenal glands were isolated and incubated in Dulbecco's Modified Eagle Medium/Nutrient Mixture F12 (0.1% BSA) (DMEM/F12 (0.1% BSA)) (basal medium) for 4 h at 37°C with gentle shaking (300 rpm), whereafter medium was collected and adrenal glands were subsequently incubated overnight in DMEM/F12 (0.1% BSA) medium containing ACTH (100 nM) (MyBioSource). Afterwards, post-incubation medium was collected and delta CORT concentrations (CORT post-incubation medium – CORT basal incubation medium) were measured with a commercially available ELISA (DRG).

### 2.2 Statistical analyses

Data are presented as box and whisker plots with median, interquartile range (25th-75th percentiles) and 10th and 90th percentiles. Differences between groups were analyzed with use of Mann-Whitney U, Student's *t*-test or Fisher exact test, as appropriate. A P-value ≤0.05 was considered statistically significant. No corrections for multiple comparisons were performed. Statistical analyses were performed with JMP Pro 17.0.0 (SAS Institute Inc., Cary, NC, USA; RRID:SCR_008567).

## 3 Results

### 3.1 The impact of cholesterol supplementation on survival and illness severity in prolonged septic mice

The five-day survival rate was comparable between LDL-cholesterol (86%) (19/22) and placebo infused septic mice (82%) (18/22) (P = 0.7) (Fig. 1A). In addition, cumulative pain scores and the rise in plasma TNF-α concentrations during prolonged sepsis (P < 0.0001 versus healthy controls) were similar in LDL-cholesterol and placebo septic mice (Fig. 1B and C). Survival of HDL-cholesterol infused septic mice (63%) (15/24) was not significantly different from placebo (84%) (16/19) on day five of sepsis-induced critical illness (P = 0.1), but was lower as compared to healthy controls (100%) (16/16) (P < 0.05) (Fig. 1D). Cumulative pain scores were higher in HDL-cholesterol infused septic mice versus placebo (P = 0.01), whereas no significant differences were observed in plasma TNF-α concentrations between HDL-cholesterol and placebo infused septic mice (P = 0.1) (Fig. 1E and F).

**Fig. 1:**
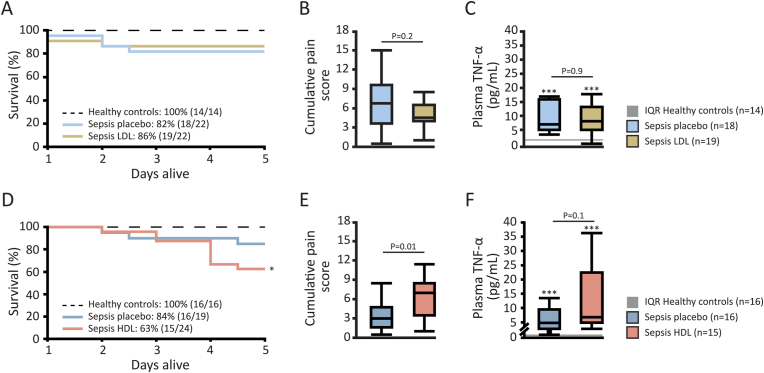
**Impact of cholesterol supplementation on survival and illness severity in prolonged septic mice.** Mouse study 1: A Kaplan-Meier survival curves of healthy control mice (n = 14/14), placebo-infused septic mice (n = 18/22) and LDL-cholesterol infused septic mice (n = 19/22) for the five-day study period. B Cumulative illness severity scores of LDL-cholesterol and placebo infused septic mice. C Plasma TNF-α concentrations. Mouse study 2: D Kaplan-Meier survival curves of healthy control mice (n = 16/16), placebo-infused septic mice (n = 16/19) and HDL-cholesterol infused septic mice (n = 15/24) for the five-day study period. E Cumulative illness severity scores of HDL-cholesterol and placebo-infused septic mice. F Plasma TNF-α concentrations. Data presented as box and whisker plots with median, IQR and 10th and 90th percentiles. Gray area represents IQR of the healthy controls. ∗/∗∗/∗∗∗P ≤ 0.05/P ≤ 0.01/P ≤ 0.0001 compared to healthy control mice. HDL, high-density lipoprotein; LDL, low-density lipoprotein; IQR, interquartile range; n, sample size.

### 3.2 The impact of cholesterol supplementation on plasma cholesterol, apolipoproteins, CORT, and on the *ex vivo* adrenal CORT response to ACTH in prolonged septic mice

Continuous infusion with LDL-cholesterol in prolonged septic mice did not affect the sepsis-induced (P < 0.05 versus healthy controls) low plasma HDL-cholesterol concentrations (P = 0.5) and plasma ApoA1 protein expression (P = 0.08), the main apolipoprotein present in HDL-cholesterol, but substantially increased plasma LDL-cholesterol concentrations (P < 0.0001) as compared to placebo (Fig. 2A–C). Plasma ApoB100 protein expression was unaffected (Fig. 2D). Whereas LDL-cholesterol infusion did not affect the sepsis-induced rise (P < 0.0001 versus healthy controls) in plasma CORT (P = 0.6), adrenocortical cholesterol depletion was attenuated compared to placebo (P = 0.0004) (Fig. 2E and F).

**Fig. 2:**
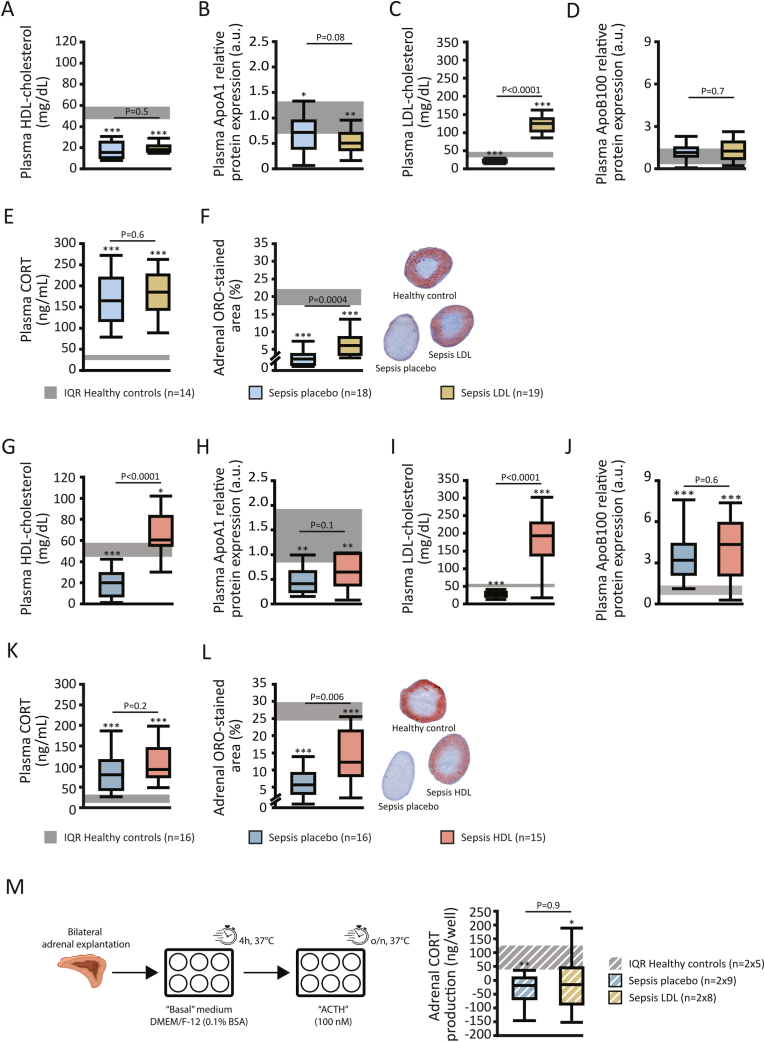
**Impact of cholesterol supplementation on plasma cholesterol, apolipoproteins, CORT, and on the adrenal CORT response to ACTH in prolonged septic mice.** Mouse study 1: A Plasma HDL-cholesterol concentrations. B Relative protein expression of plasma ApoA1. C Plasma LDL-cholesterol concentrations. D Relative protein expression of plasma ApoB100. E Plasma CORT concentrations. F Adrenal lipid content, represented as the relative amount of redness in the adrenal cortex. The right panel shows representative images for each group. Mouse study 2: G Plasma HDL-cholesterol concentrations. H Relative protein expression of plasma ApoA1. I Plasma LDL-cholesterol concentrations. J Relative protein expression of plasma ApoB100. K Plasma CORT concentrations. L Adrenal lipid content, represented as the relative amount of redness in the adrenal cortex. The right panel shows representative images for each group. Mouse study 3: M CORT increase measured in the incubation medium of explanted adrenal glands after overnight stimulation with ACTH (100 nM). Data presented as box and whisker plots with median, IQR and 10th and 90th percentiles. Gray area represents IQR of the healthy controls. Original, uncropped Western blot images for plasma ApoA1 and ApoB100 are presented in Supplemental Fig. S1. ∗/∗∗/∗∗∗P ≤ 0.05/P ≤ 0.01/P ≤ 0.0001 compared with healthy control mice. HDL, high-density lipoprotein; LDL, low-density lipoprotein; Apo, apolipoprotein; CORT, corticosterone; ACTH, adrenocorticotropic hormone; ORO, Oil-Red O; DMEM/F-12 (0.1% BSA), Dulbecco's Modified Eagle Medium/Nutrient Mixture F-12 (0.1% bovine serum albumin); IQR, interquartile range; n, sample size; a.u., arbitrary unit.

Prolonged HDL-cholesterol infusion in septic mice substantially increased plasma HDL-cholesterol concentrations (P < 0.0001) as compared to placebo, but did not further affect the reduction in plasma ApoA1 protein expression (P = 0.1) (Fig. 2G and H). In addition, plasma LDL-cholesterol concentrations were substantially increased after HDL-cholesterol infusion (P < 0.0001), whereas plasma ApoB100 protein expression remained unaffected (P = 0.6) as compared to placebo mice (Fig. 2I and J). HDL-cholesterol infusion did not affect the sepsis-induced rise (P < 0.0001 versus healthy controls) in plasma CORT (P = 0.2), though, similar as LDL-cholesterol, attenuated the depletion in adrenocortical cholesterol content (P = 0.006) versus placebo (Fig. 2K and L). However, CORT concentrations of ACTH-stimulated adrenal explants were similar for LDL-cholesterol and placebo-infused septic mice (P = 0.8) (Fig. 2M).

### 3.3 The impact of cholesterol supplementation on markers of cholesterol homeostasis and inflammation in the adrenal gland, and on adrenocortical structure in prolonged septic mice

Cholesterol can be taken up by the adrenal cortex from circulating LDL- or HDL-cholesterol lipoproteins through the LDL-R and SR-BI, respectively, or can be synthesized *de novo* within the adrenal gland. LDL- or HDL-cholesterol infusion in prolonged septic mice attenuated the sepsis-induced increase (P < 0.0001 versus healthy controls) in *Ldlr* expression as compared to placebo (P < 0.05), whereas *Scarb1* expression remained similarly increased (P < 0.0001 versus healthy controls) (Fig. 3A). Gene expression of enzymes and regulators of endogenous cholesterol synthesis (*Hmgcs1*, *Hmgcr*, *Fdft1*, *Srebf2*) were overall increased during sepsis and attenuated by LDL- or HDL-cholesterol infusion in septic mice (P < 0.05) versus placebo (Fig. 3A). Hormone sensitive lipase (*Lipe)* hydrolyzes stored cholesteryl esters to release free cholesterol for *de novo* cortisol (humans) or CORT (mice) synthesis. A similar reduction in *Lipe* (P < 0.01 versus healthy controls) was observed in LDL- or HDL-cholesterol infused septic mice, whereas key expression of markers of steroidogenesis (*StAR*, *Cyp11a1*) were overall upregulated during sepsis, unaffected by LDL- or HDL-cholesterol infusion versus placebo (Fig. 3A). No differences in *Tnf-α* mRNA expression were observed in the adrenal gland between LDL- or HDL-cholesterol and placebo infused septic mice (Fig. 3A).

**Fig. 3:**
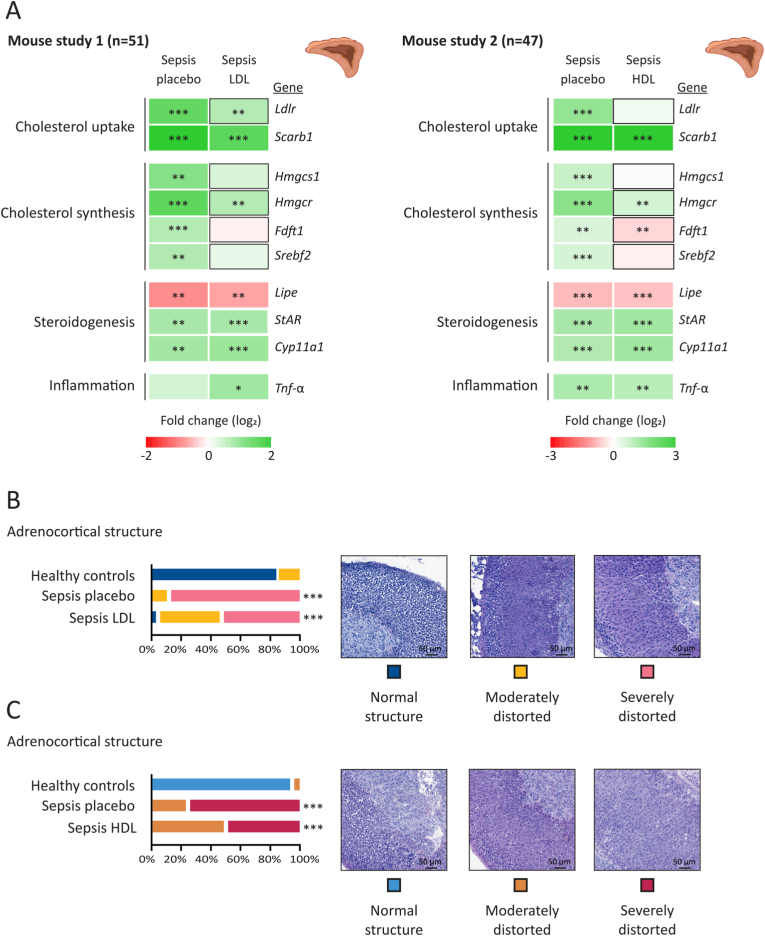
**Impact of cholesterol supplementation on markers of cholesterol homeostasis and inflammation in the adrenal gland, and on adrenocortical structure in prolonged septic mice.** A Heatmap presenting relative mRNA expression of markers of cholesterol uptake (*Ldlr*, *Scarb1*), synthesis (*Hmgcs1*, *Hmgcr*, *Fdft1*, *Srebf2*), steroidogenesis (*Lipe*, *StAR*, *Cyp11a1*) and inflammation (*Tnf-α*) in the adrenal gland. Green and red colors indicate an upregulation or downregulation in mRNA expression compared to healthy controls, respectively. Borders indicate a significant difference with the sepsis placebo group. Gene expression data are normalized to *Sdha* housekeeping gene. Mouse study 1: B Semi-quantitative scoring of adrenocortical structure, scored as either “normal structure”, “moderately distorted” or “severely distorted”. Right panels show representative images for each condition. Mouse study 2: C Semi-quantitative scoring of adrenocortical structure, scored as either “normal structure”, “moderately distorted” or “severely distorted”. Right panels show representative images for each condition. Mouse study 1: Healthy controls (n = 14); sepsis placebo (n = 18); sepsis LDL (n = 19). Mouse study 2: Healthy controls (n = 16); sepsis placebo (n = 16); sepsis HDL (n = 15). ∗/∗∗/∗∗∗P ≤ 0.05/P ≤ 0.01/P ≤ 0.0001 compared with healthy control mice. HDL, high-density lipoprotein; LDL, low-density lipoprotein; n, sample size.

We next investigated the impact of cholesterol supplementation on adrenal integrity. Both LDL- and HDL-cholesterol infusion did not reverse the sepsis-induced loss of normal adrenocortical structure as compared with placebo (P > 0.05) (Fig. 3B and C).

### 3.4 The impact of cholesterol supplementation on muscle wasting and weakness, markers of cholesterol homeostasis, and on muscle mevalonate pathway metabolites in prolonged septic mice

Septic mice receiving LDL-cholesterol infusion suffered from a similar reduction (P < 0.01 versus healthy controls) in total body weight as compared to placebo (P = 0.2) (Fig. 4A). In addition, the sepsis-induced reduction (P < 0.0001) of the tibialis anterior muscle mass was not affected by LDL-cholesterol infusion (P = 0.5), and EDL absolute and specific muscle force were equally reduced (P > 0.05) versus placebo (Fig. 4B–D). Similarly, continuous HDL-cholesterol infusion in septic mice did not further affect total body weight loss and tibialis anterior muscle mass, nor did it affect EDL absolute and specific muscle force versus placebo (P > 0.05) (Fig. 4E–H).

**Fig. 4:**
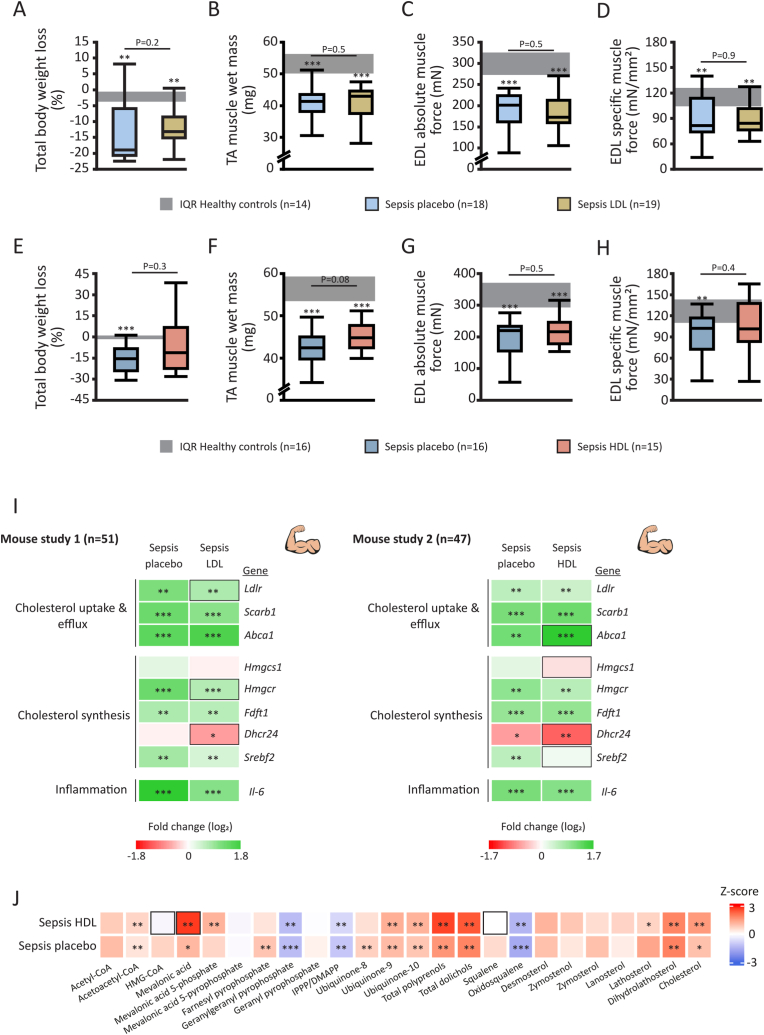
**Impact of cholesterol supplementation on muscle wasting and weakness, markers of cholesterol homeostasis and on mevalonate pathway metabolites in prolonged septic mice.** Mouse study 1: A Change in total body weight loss over five days. B Tibialis anterior wet muscle mass. C EDL absolute tetanic muscle force. D EDL specific muscle force. Mouse study 2: E Change in total body weight loss over five days. F Tibialis anterior wet muscle mass. G EDL absolute tetanic muscle force. H EDL specific muscle force. I Heatmap presenting relative mRNA expression of markers of cholesterol uptake (*Ldlr*, *Scarb1*), efflux (*Abca1*), synthesis (*Hmgcs1*, *Hmgcr*, *Fdft1*, *Dhcr24, Srebf2)* and inflammation (*Il-6*) in the skeletal muscle for mouse study 1 and mouse study 2. Green and red colors indicate an upregulation or downregulation in mRNA expression compared to healthy controls, respectively. Borders indicate a significant difference with the sepsis placebo group. Gene expression data are normalized to *Gapdh* housekeeping gene. J Mevalonate pathway metabolites in the skeletal muscle of mice from mouse study 2, and quantified with LC-MS and presented as mean Z-scores of the healthy controls. Borders indicate a significant difference with placebo septic mice. Data presented as box and whisker plots with median, IQR and 10th and 90th percentiles. Gray area represents IQR of the healthy controls. Mouse study 1: Healthy controls (n = 14); sepsis placebo (n = 18); sepsis LDL (n = 19). Mouse study 2: Healthy controls (n = 16); sepsis placebo (n = 16); sepsis HDL (n = 15). ∗/∗∗/∗∗∗P ≤ 0.05/P ≤ 0.01/P ≤ 0.0001 compared with healthy control mice. HDL, high-density lipoprotein; LDL, low-density lipoprotein; TA, tibialis anterior; EDL, extensor digitorum longus; LC-MS, Liquid-Chromatography Mass Spectrometry; IQR, interquartile range; n, sample size.

We next investigated gene expression of markers of cholesterol uptake, efflux and synthesis in the skeletal muscle of prolonged septic mice (Fig. 4I). LDL-, but not HDL-cholesterol, infusion attenuated the sepsis-induced increase in *Ldlr* expression (P < 0.05) versus placebo, without further affecting *Scarb1* expression (Fig. 4I). As compared with placebo, LDL-cholesterol infusion did not affect the sepsis-induced rise in *Abca1* (P < 0.01 versus healthy controls), whereas its expression was further increased by HDL-cholesterol infusion (P = 0.01) (Fig. 4I). In addition, as compared with placebo, LDL- or HDL-cholesterol infusion moderately decreased gene expression of markers of cholesterol synthesis in the muscle: LDL-cholesterol (*Hmgcr* (P = 0.03); *Dhcr24* (P = 0.04)); HDL-cholesterol (*Hmgcs1* (P = 0.02); *Dhcr24* (P = 0.01); *Srebf2* (P = 0.04)) (Fig. 4I). Also, mRNA expression of *Il-6* was similarly increased (P < 0.0001 versus healthy controls) in LDL- or HDL-cholesterol and placebo-infused septic mice (Fig. 4I).

Next, mevalonate pathway metabolites were quantified in the skeletal muscle (Fig. 4J). HDL-cholesterol infusion moderately further increased the sepsis-induced rise (P < 0.05 versus healthy controls) in muscle mevalonic acid concentrations (P = 0.02), but did not affect farnesyl pyrophosphate concentrations, and only moderately decreased HMG-CoA concentrations in the skeletal muscle (P = 0.02) as compared to placebo (Fig. 4J). In addition, sterol intermediates (squalene, desmosterol, lanosterol, lathosterol) were unaffected in the muscle of placebo septic mice and were either not, or moderately affected with HDL-cholesterol infusion (squalene: P = 0.008 versus placebo). Similarly, compared with placebo, HDL-cholesterol infusion did not affect the sepsis-induced increase (P < 0.05 versus healthy controls) in skeletal muscle cholesterol concentrations (Fig. 4J). In addition, muscle ubiquinone-9, the dominant CoQ enzyme in rodents involved in the electron transport chain, and total dolichol concentrations, both nonsterol isoprenoids, were increased during prolonged sepsis (P < 0.01 versus healthy controls), but not further affected by HDL-cholesterol infusion (Fig. 4J).

### 3.5 The impact of cholesterol supplementation on markers of cholesterol and bile acid homeostasis in the liver, and on hepatic structure in prolonged septic mice

After five days of sepsis-induced critical illness, liver wet weight of placebo infused mice was decreased as compared with healthy controls (P < 0.01), but was significantly increased after LDL-cholesterol infusion (P = 0.001) versus placebo (Fig. 5A). Histologically, septic mice receiving LDL-cholesterol infusion displayed a similar distortion in hepatic structure versus placebo (P = 0.4) (Fig. 5B). As compared with placebo, LDL-cholesterol infusion moderately increased plasma total bile acid concentrations (P = 0.05) (Fig. 5C). HDL-cholesterol infusion did not affect the sepsis-induced reduction in liver weight (P = 0.1), nor did it affect the loss of hepatic structure compared to placebo (P = 0.5) (Fig. 5D and E). Plasma total bile acid concentrations were also moderately increased in HDL-cholesterol infused septic mice versus placebo (P = 0.04) (Fig. 5F).

**Fig. 5:**
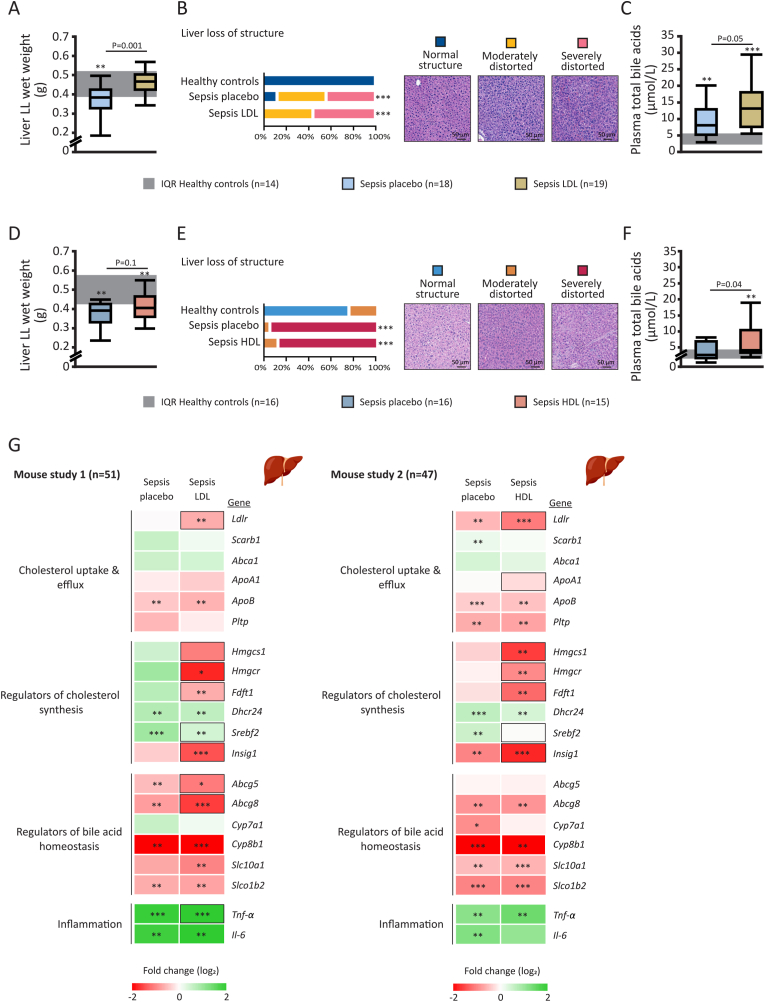
**Impact of cholesterol supplementation on markers of cholesterol and bile acid homeostasis in the liver, and on hepatic structure during prolonged sepsis.** Mouse study 1: A Liver LL wet weight. B Semi-quantitative scoring of liver structure, scored as either “normal structure”, “moderately distorted” or “severely distorted”. Right panels show representative images for each condition. C Plasma total bile acid concentrations. Mouse study 2: D Liver LL wet weight. E Semi-quantitative scoring of liver structure, scored as either “normal structure”, “moderately distorted” or “severely distorted”. Right panels show representative images for each condition. F Plasma total bile acid concentrations. G Heatmap presenting relative mRNA expression of markers of cholesterol uptake and efflux, regulators of cholesterol and bile acid synthesis, homeostasis and inflammation in the liver for mouse study 1 and mouse study 2. Green and red colors indicate an upregulation or downregulation in mRNA expression compared to healthy controls, respectively. Borders indicate a significant difference with the sepsis placebo group. Gene expression data are normalized to *Rn18S* housekeeping gene. Data presented as box and whisker plots with median, IQR and 10th and 90th percentiles. Gray area represents IQR of the healthy controls. Mouse study 1: Healthy controls (n = 14); sepsis placebo (n = 18); sepsis LDL (n = 19). Mouse study 2: Healthy controls (n = 16); sepsis placebo (n = 16); sepsis HDL (n = 15). ∗/∗∗/∗∗∗P ≤ 0.05/P ≤ 0.01/P ≤ 0.0001 compared with healthy control mice. HDL, high-density lipoprotein; LDL, low-density lipoprotein; LL, left lobe; IQR, interquartile range; n, sample size.

We next investigated gene expression of markers of cholesterol homeostasis in the liver. Five-day LDL- or HDL-cholesterol infusion in septic mice attenuated hepatic *Ldlr* expression (P < 0.05) versus placebo, without affecting *Scarb1* and *Abca1* (Fig. 5G). Gene expression of *ApoA1* was moderately decreased by HDL-cholesterol infusion versus placebo (P = 0.04), whereas the sepsis-induced reduction in *ApoB* was not affected by LDL- or HDL-cholesterol infusion (Fig. 5G). Phospholipid transfer protein (*Pltp*), mediating HDL particle remodeling and phospholipid transfer between lipoproteins, was not differently expressed at the mRNA level with LDL- or HDL-cholesterol infusion as compared with placebo (Fig. 5G). Markers of endogenous cholesterol synthesis (*Hmgcs1*, H*mgcr*, *Fdft1*) were overall decreased in the liver of prolonged septic mice receiving LDL- and HDL-cholesterol infusion versus placebo (P < 0.05) (Fig. 5G). Also, the sepsis-induced increase in gene expression of *Srebf2*, a transcriptional regulator of cholesterol synthesis, was attenuated in LDL- or HDL-cholesterol infusion versus placebo (P < 0.05), in addition to *Insig1*, mediating feedback inhibition of sterol regulatory element-binding protein (SREBP)-regulated cholesterol synthesis, of which the expression was also decreased (P < 0.05) (Fig. 5G).

Markers of biliary cholesterol transport (*Abcg5*, *Abcg8*), bile acid synthesis (*Cyp8b1*), and bile acid uptake transport (*Slco1b2*) were overall decreased during prolonged sepsis, and moderately further decreased by LDL-cholesterol infusion (*Abcg5*: P = 0.03; *Abcg8*: P = 0.04) versus placebo (Fig. 5G). An increased expression of *Tnf-α* was observed in the liver during prolonged sepsis (P < 0.01 versus healthy controls), which was further increased by LDL-cholesterol infusion (P = 0.004), but unaffected by HDL-cholesterol (P = 0.7), as compared with placebo (Fig. 5G).

## 4 Discussion

We here demonstrated that supplementation with either an LDL- or HDL-cholesterol mixture reversed hypocholesterolemia in a mouse model of prolonged sepsis, whereas this did not improve the sepsis-induced adrenal or muscle phenotype. Continuous infusion with LDL- or HDL-cholesterol increased adrenocortical lipid content, though failed to improve the *ex vivo* blunted CORT response to ACTH in prolonged septic mice, and did also not affect the sepsis-induced loss of muscle mass and force.

A rapid and sustained reduction in plasma LDL- and HDL-cholesterol has been observed during sepsis, associated with poor outcome (Hofmaenner et al., 2023; Tanaka et al., 2017; Chiarla et al., 2010; Giovannini et al., 1999; Dunham et al., 2003). We here documented that continuous infusion with a cholesterol mixture enriched in LDL-cholesterol substantially increased plasma LDL-cholesterol concentrations in prolonged septic mice, without affecting plasma HDL-cholesterol, whereas infusion with an HDL-cholesterol enriched mixture increased plasma HDL- and even more so LDL-cholesterol. In the current studies, no survival benefit from LDL- or HDL-cholesterol infusion was observed in our fluid-resuscitated, antibiotics-treated mouse model of prolonged sepsis, which might differ from treatment effects in an acute, high-mortality context (Tanaka et al., 2020; Dai et al., 2010). Variable purity and composition of cholesterol and lipoprotein mixtures can also complicate clinical translatability and might at least partially explain inconsistent outcome benefits in (pre) clinical studies (Levine et al., 1993).

The differential effect of LDL- and HDL-cholesterol supplementation on endogenous plasma lipoproteins in the current study might also be due to compositional differences in the administered commercial cholesterol mixtures or result from lipoprotein remodeling. Lipid metabolism significantly differs between species, and therefore, extrapolations from mice to humans should be done with caution (Yin et al., 2012). In contrast to humans, mice have higher plasma HDL-cholesterol concentrations, lower plasma LDL-cholesterol concentrations, and preferentially utilize SR-BI-mediated cholesterol uptake as the dominant source of cholesterol supply for steroidogenesis (Yin et al., 2012; Kraemer, 2007). Moreover, mice naturally lack cholesterol ester transfer protein (CETP) and lipoprotein(a) (Lp(a)) (Gordon et al., 2015). In addition, lower LDL-receptor binding affinity (Corsini et al., 1992), together with more rapid HDL remodeling and clearance may influence cholesterol-associated lipoprotein distribution and cholesterol handling (Zannis et al., 2015). Indeed, in mice, HDL particles can redistribute cholesterol towards ApoB-containing lipoproteins through CETP-independent mechanisms including PLTP and lecithin-cholesterol acyltransferase (LCAT) (Zannis et al., 2015), which might at least to some extent also explain the increase in plasma LDL-cholesterol with HDL-cholesterol infusion. Furthermore, the observed dissociation between the affected plasma LDL-cholesterol concentrations but unchanged ApoB100 protein expression may reflect changes in lipoprotein size, composition and kinetics rather than in the number of ApoB-containing particles. Smaller, more cholesterol-depleted ApoB-containing particles may circulate longer in circulation due to reduced LDL-receptor affinity. In contrast, the rapid turnover and remodeling of HDL lipoprotein particles may, at least partially, explain the reduction in both circulating HDL-cholesterol concentrations and ApoA1 protein expression levels (Zannis et al., 2015).

A sustained low cholesterol availability could become disadvantageous for the adrenal gland, especially in the prolonged phase of illness, because this could impair stress-responsiveness (van der Voort et al., 2003). We here demonstrated that LDL- or HDL-cholesterol infusion in prolonged septic mice partially reversed the loss of adrenal cholesterol content, without affecting plasma CORT concentrations. Although LDL- and HDL-cholesterol infusion improved the sepsis-induced depletion in adrenocortical cholesterol content, markers of steroidogenesis remained unaffected, and *ex vivo* overnight stimulation with ACTH did also not increase CORT secretion of explanted adrenal glands. A similar finding was previously documented in an experimental setup of five-day continuous hydrocortisone infusion which was also able to counteract adrenal cholesterol depletion (De Bruyn et al., 2024). The lack of improved CORT responsiveness might be due to impaired lipid droplet handling, or impaired ACTH signaling and steroidogenesis (Boonen et al., 2013, 2014; Kraemer, 2007). Emerging evidence points to a role for cholesterol in controlling muscle function through its role in membrane integrity and signal transduction (Goossens et al., 2021; Barrientos et al., 2015). An altered cholesterol availability has been involved in the development of ICUAW (Goossens et al., 2021), and statin treatment has been shown to associate with myopathy in a non-critically ill context (Nguyen et al., 2018). We here documented that increased circulating cholesterol availability with use of LDL- or HDL-cholesterol infusion did not affect the loss of muscle mass and force but also did not affect myofiber cholesterol content in prolonged septic mice, irrespective of any effect on muscle structure. Why myofiber cholesterol content was not increased by the supplementation, in contrast to the increase in cholesterol in adrenal tissue, is not clear. Mitochondrial dysfunction may additionally contribute to the pathophysiology of adrenal and muscle dysfunction during prolonged critical illness through disturbances in oxidative metabolism, impaired mitochondrial cholesterol trafficking, and steroidogenesis (Miller, 2007; Uzun et al., 2025). Of note, mitochondrial damage also seems to be one of the main pathological findings in sepsis-induced cardiomyopathy (Jacobs et al., 2025; Smeding et al., 2012; Wu et al., 2024), but was beyond the scope of the present study. Remarkably, in a clinically relevant, acute septic rat model, HDL-cholesterol treatment reversed the sepsis-induced reduction in cardiomyocyte membrane cholesterol but did not affect the reduction in liver membrane cholesterol (Kleyman et al., 2025). We observed a marked decrease in markers of cholesterol synthesis in the liver, adrenal gland, and skeletal muscle upon treatment with either LDL- or HDL-cholesterol, likely reflecting feedback inhibition in response to prolonged LDL- or HDL-cholesterol infusion. Indeed, the evolutionary conserved and tightly regulated mechanisms of cholesterol homeostasis appear to counteract therapeutic strategies investigating cholesterol supplementation in sepsis. In contrast to cholesterol, which primarily serves structural and steroidogenic functions, 3HB supplementation in prolonged septic mice was associated with enhanced intramuscular uptake of 3HB and subsequent ketone-to-mevalonate metabolism, which was associated with improved muscle function (Goossens et al., 2021). Moreover, ketone bodies appear to be preferentially utilized as signaling molecules rather than energy substrates in septic mice, suggesting that the beneficial effects of 3HB on muscle function may not solely depend on restoration of cholesterol substrate availability, but additionally involved activation of downstream metabolic pathways, although the exact mechanisms remain unclear (Goossens et al., 2019).

This study has some limitations. First, translation from the mouse model of prolonged sepsis-induced critical illness to the human setting must be done with caution, as septic mice were not mechanically ventilated nor fully immobilized, but did receive intravenous fluid resuscitation, nutritional support, and antibiotics and analgesics to mimic the clinical setting as much as possible. Second, mice typically have lower plasma LDL-cholesterol concentrations as compared to humans and lack the CETP enzyme (Yin et al., 2012). To partially account for species-specific differences in lipoprotein distribution and cholesterol handling, supplementation with both LDL- and HDL-cholesterol was investigated in mice. Nevertheless, extrapolation regarding the relative contribution of the different lipoproteins in humans must be done with caution. Third, markers of cholesterol homeostasis were mainly investigated at the gene expression level, which might not always reflect protein change. In addition, mitochondrial function was not directly assessed, limiting mechanistic understanding of potential downstream metabolic alterations contributing to adrenal and muscle dysfunction during prolonged sepsis. Fourth, the cholesterol preparations used for these experiments were derived from bovine (LDL-infusion) or human (HDL-infusion) sources and may have affected host responses, although we did not observe a shift in mortality or in cytokine profiles.

Our findings demonstrated the feasibility to reverse hypocholesterolemia in a preclinical mouse model of sepsis, but do not support a protective effect on adrenal failure or muscle weakness. Novel adjunctive treatment options for either of these debilitating complications of critical illness are still needed will likely have a major impact on clinical outcome. Although cholesterol is essential for steroidogenesis, our findings suggest that factors beyond substrate availability contribute to adrenal and muscle dysfunction. Future studies are needed to further elucidate downstream intracellular mechanisms, including altered cholesterol trafficking and lipid handling, and impaired ACTH signaling and steroidogenesis. In addition, careful evaluation of the optimal composition, efficacy, dosing, and safety of lipoprotein-based therapeutic approaches is needed prior to any clinical translation.

## 5 Conclusion

LDL- and HDL-cholesterol supplementation reversed hypocholesterolemia in prolonged septic mice, though without reversing the adrenal or muscle sepsis phenotype. In combination with the suppressed markers of cholesterol uptake and synthesis and increased bile acid formation these findings argue against the need to treat hypocholesterolemia in prolonged sepsis.

## Ethical approval

The Animal Ethics Committee of the KU Leuven approved the protocol for the mouse study (P098/2021). The study adheres to the European Union concerning the welfare of laboratory animals as declared in Directive (2010)/63/EU and complied with the ARRIVE guidelines.

## Consent for publication

Not applicable.

## Funding

This work was supported by the Methusalem program of the Flemish Government (through the KU Leuven to GVdB and LL, METH/14/06); by the European Research Council Advanced Grants (AdvG-2017-785806 and AdvG-2023-101133276) to GVdB, and by the Research Foundation – Flanders, Belgium (grant G069421N to LL and GVdB).

## CRediT authorship contribution statement

**Lauren De Bruyn:** Conceptualization, Data curation, Formal analysis, Investigation, Methodology, Project administration, Software, Validation, Visualization, Writing – original draft, Writing – review & editing. **Fien Van Beek:** Formal analysis, Investigation. **Sarah Vander Perre:** Investigation. **Sarah Derde:** Investigation. **Inge Derese:** Investigation. **Lies Pauwels:** Investigation. **Greet Van den Berghe:** Conceptualization, Funding acquisition, Methodology, Project administration, Resources, Supervision, Validation, Writing – original draft, Writing – review & editing. **Lies Langouche:** Conceptualization, Data curation, Formal analysis, Funding acquisition, Methodology, Project administration, Resources, Supervision, Validation, Writing – original draft, Writing – review & editing.

## Declaration of competing interest

The authors declare that they have no known competing financial interests or personal relationships that could have appeared to influence the work reported in this paper.

## Data Availability

Some or all datasets generated during and/or analyzed during the current study are not publicly available but are available from the corresponding author on reasonable request. Data are located in controlled access data repository of the KU Leuven.

## Abbreviations:

ACTH: Adrenocorticotropic hormone
Apo: Apolipoprotein
BSA: Bovine serum albumin
CETP: Cholesterol ester transfer protein
CORT: Corticosterone
DMAPP: Dimethylallyl pyrophosphate
DMEM/F12: Dulbecco's Modified Eagle Medium/Nutrient Mixture F12
EDL: Extensor digitorum longus
ELISA: Enzyme-linked immunosorbent assay
3HB: 3-hydroxybutyrate
HRP: Horseradish peroxidase
HDL: High-density lipoprotein
ICU: Intensive care unit
ICU-AI: ICU-acquired adrenal insufficiency
ICUAW: ICU-acquired weakness
IPPP: Isopentenyl pyrophosphate
LCAT: Lecithin-cholesterol acyltransferase
LC-MS: Liquid-chromatography mass spectrometry
LDL: Low-density lipoprotein
PLTP: Phospholipid transfer protein
PVDF: Polyvinylidene fluoride
SREBP: Sterol regulatory element-binding protein
VLDL: Very-low density lipoprotein
