# Supplementary material for: Revisiting hypocholesterolemia during prolonged sepsis: From targeted cholesterol repletion to unresolved adrenal and muscle dysfunction
Source: Mol Cell Endocrinol. 2026 Oct;620:112848. doi: 10.1016/j.mce.2026.112848 (PMC13384042; doi:10.1016/j.mce.2026.112848)
Supplement: Supplementary file 2 [file mmc1.docx]

**Revisiting hypocholesterolemia during prolonged sepsis: From targeted cholesterol repletion to unresolved adrenal and muscle dysfunction**

Lauren De Bruyn^1^, Fien Van Beek^1^, Sarah Vander Perre^1^, Sarah Derde^1^, Inge Derese^1^, Lies Pauwels^1^, Greet Van den Berghe^1^*, Lies Langouche^1^*

* contributed equally

**Affiliations:** ^1^ Clinical Division and Laboratory of Intensive Care Medicine, Department of Cellular and Molecular Medicine, KU Leuven, 3000 Leuven, Belgium.

**Supplemental File**

**Supplemental Table S1**

**Table S1: List of used gene expressions**

| Gene symbol  (Mus Musculus) | Gene name | Product ID  (Thermofisher Scientific) |
| --- | --- | --- |
| *Abca1* | ATP-binding cassette subfamily A member 1 | Mm00442646_m1 |
| *Abcg5* | ATP-binding cassette subfamily G member 5 | Mm00446241_m1 |
| *Abcg8* | ATP-binding cassette subfamily G member 8 | Mm00445980_m1 |
| *ApoA1* | Apolipoprotein A1 | Mm00437569_m1 |
| *ApoB* | Apolipoprotein B | Mm01545150_m1 |
| *Cyp7a1* | Cholesterol 7 alpha-hydroxylase | Mm00484150_m1 |
| *Cyp11a1* | Cholesterol side-chain cleavage enzyme | Mm00490735_m1 |
| *Cyp8b1* | Cytochrome P450, family 8, subfamily B, polypeptide 1 | Mm00501637_s1 |
| *Dhcr24* | 24-Dehydrocholesterol reductase | Mm00519071_m1 |
| *Fdft1* | Farnesyl-diphosphate farnesyltransferase 1 | Mm01598574_g1 |
| *Gapdh* | Glyceraldehyde 3-phosphate dehydrogenase | 4352339E |
| *Hmgcr* | 3-Hydroxy-3-methylglutaryl-coA reductase | Mm01282499_m1 |
| *Hmgcs1* | 3-Hydroxy-3-methylglutaryl-CoA synthase 1 | Mm01304569_m1 |
| *Insig1* | Insulin-induced gene 1 | Mm00463389_m1 |
| *Il-6* | Interleukin 6 | Mm00446190_m1 |
| *Ldlr* | Low-density lipoprotein receptor | Mm00440166_g1 |
| *Lipe* | Hormone sensitive lipase | Mm00495359_m1 |
| *Pltp* | Phospholipid transfer protein | Mm01240573_m1 |
| *Rn18S* | 18S ribosomal RNA | Mm03928990_g1 |
| *Scarb1* | Scavenger receptor class B type 1 | Mm00450234_m1 |
| *Sdha* | Succinate dehydrogenase complex, subunit A | Mm01352366_m1 |
| *Slc10a1* | Sodium/bile acid cotransporter family member 1 | Mm00441421_m1 |
| *Slco1b2* | Solute carrier organic anion transporter family member 1B2 | Mm00451510_m1 |
| *Srebf2* | Sterol regulatory element binding factor 2 | Mm01306292_m1 |
| *Star* | Steroidogenic acute regulatory protein | Mm00441558_m1 |
| *Tnf-α* | Tumor necrosis factor alpha | Mm00443258_m1 |

**Supplemental Figure S1**

**Mouse study 1:**

**Plasma ApoA1 protein expression – uncropped WB image gel 1-4**

M: marker ; HC: Healthy control ; PbO: Sepsis placebo ; LDL: Sepsis LDL

**Plasma ApoB100 protein expression – uncropped WB image gel 1-4**

M: marker ; HC: Healthy control ; PbO: Sepsis placebo ; LDL: Sepsis LDL

**Mouse study 2:**

**Plasma ApoA1 protein expression – uncropped WB image gel 1-3**

M: marker ; HC: Healthy control ; PbO: Sepsis placebo ; HDL: Sepsis HDL

**Plasma ApoAB100 protein expression – uncropped WB image gel 1-3:**

M: marker ; HC: Healthy control ; PbO: Sepsis placebo ; HDL: Sepsis HDL
